# Supplementary material for: Annual risk of hepatitis E virus infection and seroreversion: Insights from a serological cohort in Sitakunda, Bangladesh
Source: Epidemiol Infect. 2024 Mar 18;152:e52. doi: 10.1017/S0950268824000438 (PMC11022260; doi:10.1017/S0950268824000438)
Supplement: Dighe et al. supplementary material [file S0950268824000438sup001.docx]

***Epidemiology and Infection***

Annual risk of hepatitis E virus infection and seroreversion: insights from a serological cohort in Sitakunda, Bangladesh

Amy Dighe^†1^, Ashraful Islam Khan^†2^, Taufiqur Rahman Bhuiyan^2^, Md Taufiqul Islam^2^, Zahid Hasan Khan^2^, Ishtiakul Islam Khan^2^, Juan Dent Hulse^1^, Shakeel Ahmed^3^, Mamunur Rashid^3^, Md Zakir Hossain^3^, Rumana Rashid^3^, Sonia Hegde^1^, Emily S Gurley^‡1^, Firdausi Qadri^‡2^, Andrew S Azman^‡1,4,5^

1. Johns Hopkins Bloomberg School of Public Health, Baltimore, Maryland, USA.
2. icddr,b, Dhaka, Bangladesh.
3. Bangladesh Institute of Tropical and Infectious Diseases, Chattogram, Bangladesh
4. Geneva Centre for Emerging Viral Diseases, Geneva University Hospitals, Geneva, Switzerland
5. Division of Tropical and Humanitarian Medicine, Geneva University Hospitals, Geneva, Switzerland

**Corresponding author:* [*adighe1@jhmi.edu*](mailto:adighe1@jhmi.edu)*, Johns Hopkins Bloomberg School of Public Health, 615 N Wolfe St, Baltimore, MD 21205*

*†Equal contributions – first authors*

*‡Equal contributions – last authors*

**Supplementary Materials**

**Figure S1.** Location of sampled sites.

**Catalytic model solutions**

**Figure S2.** Directed Acyclic Graph representing the causal relationships between potential risk factors and HEV seropositivity, as assumed *a priori*

**Table S1**. Number of individuals and households sampled during the study.

**Figure S3.** Semivariogram of the semivariance in household level seroprevalence.

**Table S2**: Demographic characteristics of study participants.

**Figure S4**. Annual seroconversion and seroreversion rates by age.

**Figure S5**. The changes in the optical density (od) to cut-off ratios for samples taken at baseline and at follow-up

**Table S3.** The difference in expected log predicted density (ELPD) compared to the preferred catalytic model.

**Figure S6.** Predicted seroprevalence by age.

**Figure S7.** The expected log-predictive density (ELPD) for different age thresholds for changing risk of infection

**Figure S8.** A comparison of the age-stratified seroprevalence by round.

**Table S4.** A comparison of annual risk of infection estimates when using age-stratified seroprevalence at baseline versus at follow-up.

**Figure S9.** A comparison of posterior estimates of the annual risk of infection when we fit the rate of seroreversion, ρ, simultaneously with the annual risk of infection, to age-stratified cross-sectional seroprevalence data.

**Additional References cited in the Supplemental Materials**


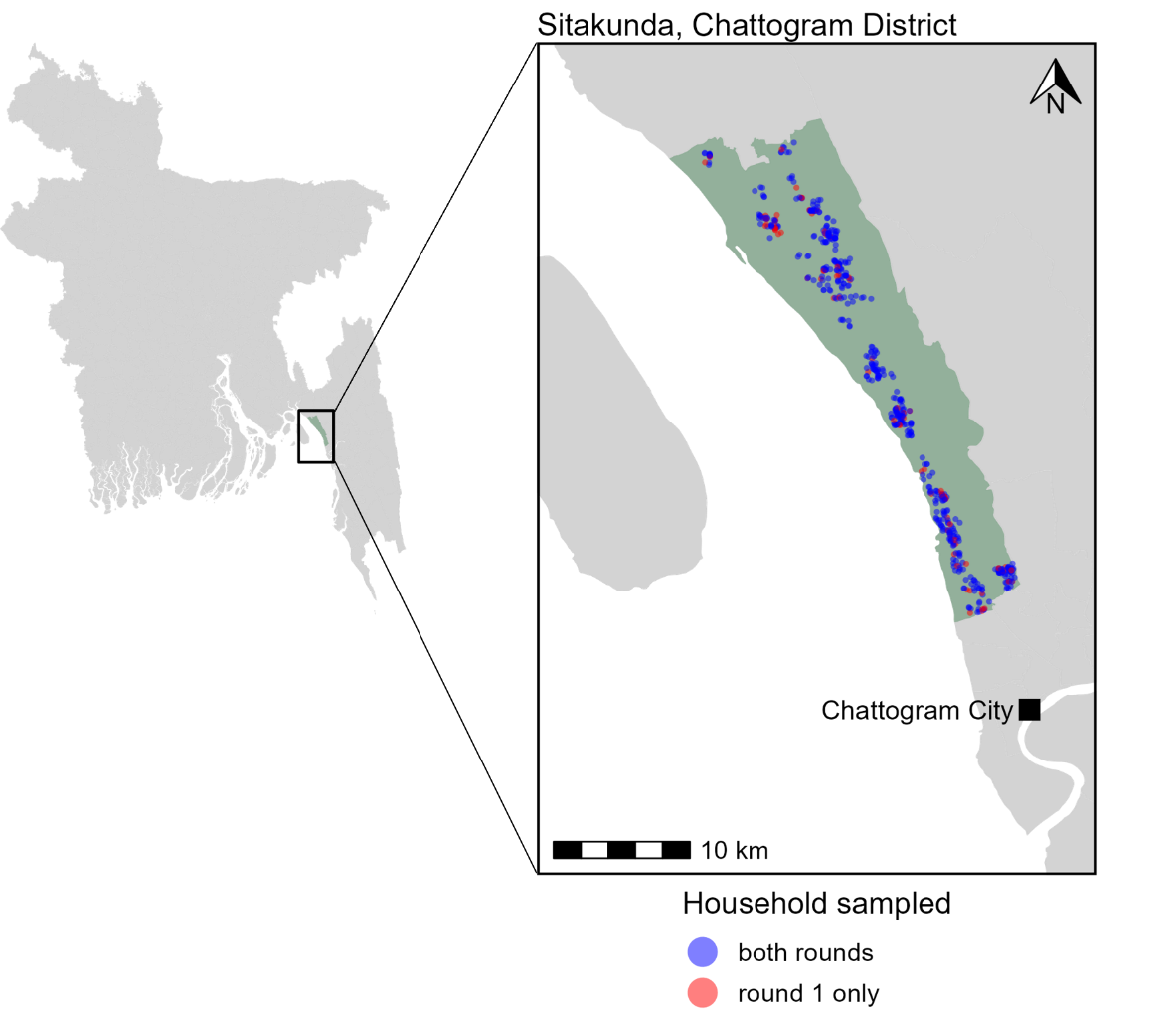


***Figure S1.*** *Location of sampled sites. Sitakunda (green) within Chattogram district, Bangladesh. Sample sites are shown on the inset map, with households sampled in both rounds represented in blue and households sampled at baseline only before being lost to follow-up represented in red.*

### **Catalytic model solutions**

1. Model 1 - without seroreversion:


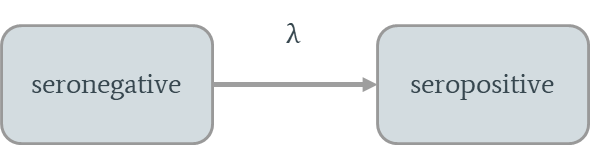


- 1. Assuming a constant force of infection, λ, with age and time, the proportion seropositive, *p*, at age *a* is:

$$p(a)=1-e^{-\lambda a}$$

And so, the proportion seropositive, *p*, in the age class spanning *a_1_-a_2_* is given by:

$$\begin{aligned} p\left( \bar{a_{1}\to a_{2}} \right)=\frac{1}{a_{2}-a_{1}} \left( {a_{2}-a_{1}+ \frac{1}{\lambda}( e}^{-\lambda a_{2}}- e^{-\lambda a_{1}}) \right) \end{aligned}$$

- 1. Assuming the force of infection can be different in children ≤a_0_ years old and adults >a_0_

1. When a ≤a_0_ the proportion seropositive at age a is:

$$p\left( a \right)={1- e}^{-\lambda_{C}a}$$

And so, the proportion seropositive in the age class spanning a_1_-a_2_ where both are ≤a_0_ is:

$$p\left( \bar{a_{1}\to a_{2}} \right)=\frac{1}{a_{2}-a_{1}} \left( {a_{2}-a_{1}+ \frac{1}{\lambda_{C}}( e}^{-\lambda_{C}a_{2}}- e^{-\lambda_{C}a_{1}}) \right)$$

1. For adults, where age a>a_0_, the proportion seropositive at age a is:

$$p\left( a \right)={1- e}^{-\lambda_{C}a_{0}- \lambda_{A}({a-a}_{0})}$$

And so, the proportion seropositive in the age class spanning a_1_-a_2_ where both are >a_0_ is:

$$p\left( \bar{a_{1}\to a_{2}} \right)=\frac{1}{a_{2}-a_{1}} \left( {a_{2}-a_{1}+ \frac{1}{\lambda_{A}}( e}^{-{(\lambda}_{C}a_{0}+\lambda_{A}{(a_{2}-a}_{0})}- e^{-{(\lambda}_{C}a_{0}+\lambda_{A}{(a_{1}-a}_{0})}) \right)$$

1. Model 2 - with seroreversion:


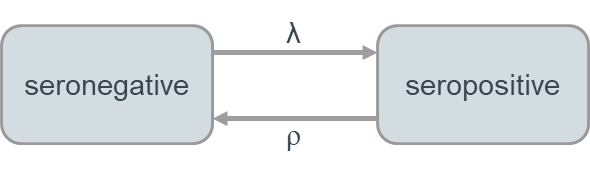


- 1. Assuming a constant force of infection, λ, with age and time, the proportion seropositive, *p*, at age *a* is:

$$p\left( a \right)=\frac{\lambda}{\lambda+\sigma} \left( {1 - e}^{-\left( \lambda+\sigma\right)a} \right)$$

And so, the proportion seropositive, *p*, in the age class spanning *a_1_-a_2_* is given below, where *σ* is the annual rate of seroreversion.

$$\begin{aligned} p\left( \bar{a_{1}\to a_{2}} \right)=\frac{1}{a_{2}-a_{1}} \left( \frac{\lambda}{\lambda+\sigma}\left( {{(a}_{2}-a_{1})+ \frac{1}{\lambda+\sigma}( e}^{-\left( \lambda+\sigma\right)a_{2}}- e^{-\left( \lambda+\sigma\right)a_{1}}) \right) \right) \end{aligned}$$

- 1. Assuming the force of infection and the annual rate of seroreversion vary with age.

The force of infection changes at age a_λ_ and the annual rate of seroreversion changes at age a_σ_. We assume a_λ_> a_σ._

- - 1. When a ≤ a_σ_: the proportion seropositive at age a is:

$$p_{1}\left( a \right)={\frac{\lambda_{I}}{\lambda_{I}+\sigma_{I}}(1- e}^{-{(\lambda}_{I}+\sigma_{I})a})$$

And so, when we solve for the age class spanning a_1_-a_2_, where both are ≤ a_σ_, we get:

$$\begin{aligned} p_{1}\left( \bar{a_{1}\to a_{2}} \right)=\frac{1}{a_{2}-a_{1}} \left( \frac{\lambda_{I}}{\lambda_{I}+\sigma_{I}}\left( {(a}_{2}-a_{1})+ \frac{1}{\lambda_{I}+\sigma_{I}}(e^{-\left( \lambda_{I}+\sigma_{I} \right)a_{2}})- e^{-\left( \lambda_{I}+\sigma_{I} \right)a_{1}}) \right) \right) \# \end{aligned}$$

- - 1. When a_σ_< a ≤ a_λ_

$$p_{2}\left( a \right)=1-\left[ \left( 1-p_{1}\left( a_{\sigma} \right) \right)*\left( 1-{\frac{\lambda_{I}}{\lambda_{I}+\sigma_{II}}(1- e}^{-{(\lambda}_{I}+\sigma_{II}){(a-a}_{\sigma})}) \right) \right]$$

And so, when we solve for the age class a_1_-a_2_, where both are >a_σ_ and ≤ a_λ_, we get:

$$p_{2}\left( \bar{a_{1}\to a_{2}} \right)=\frac{1}{a_{2}-a_{1}}\left[ {(a}_{2}-a_{1})-\left( 1-p_{1}\left( a_{\sigma} \right) \right)*\left( {(a}_{2}-a_{1})*\left( 1-\frac{\lambda_{I}}{\lambda_{I}+\sigma_{II}} \right)-\frac{1}{\lambda_{I}+\sigma_{II}}*\frac{\lambda_{I}}{\lambda_{I}+\sigma_{II}}*e^{{(\lambda}_{I}+\sigma_{II})a_{\sigma}}*(e^{-{{(\lambda}_{I}+\sigma_{II})a}_{2}}-e^{-{{(\lambda}_{I}+\sigma_{II})a}_{1}}) \right) \right]$$

- - 1. When a> a_λ_

$$p_{3}\left( a \right)=1-\left[ \left( 1-p_{2}\left( a_{\lambda} \right) \right)*\left( 1-{\frac{\lambda_{II}}{\lambda_{II}+\sigma_{II}}(1- e}^{-{(\lambda}_{II}+\sigma_{II}){(a-a}_{\lambda})}) \right) \right]$$

And so, when we solve for the age class a_1_-a_2_, where both are >a_σ_ and ≤ a_λ_, we get:

$$p_{3}\left( \bar{a_{1}\to a_{2}} \right)=\frac{1}{a_{2}-a_{1}}\left[ {(a}_{2}-a_{1})-\left( 1-p_{2}\left( a_{\lambda} \right) \right)*\left( {(a}_{2}-a_{1})*\left( 1-\frac{\lambda_{II}}{\lambda_{II}+\sigma_{II}} \right)-\frac{1}{\lambda_{II}+\sigma_{II}}*\frac{\lambda_{II}}{\lambda_{II}+\sigma_{II}}*e^{{(\lambda}_{II}+\sigma_{II})a_{\lambda}}*(e^{-{{(\lambda}_{II}+\sigma_{II})a}_{2}}-e^{-{{(\lambda}_{II}+\sigma_{II})a}_{1}}) \right) \right]$$

**Key justifications for assumed causal relationships represented in Figure S2.**

We assume that sex could modify the relationship between past HEV infection and HEV seropositivity through sex differences in humoral immune response [S1] but that sex primarily relates to HEV seropositivity through behavioural factors, including occupation and travel as well as factors we did not measure. We assume that age relates to HEV infection through time spent at risk, as well as age differences in behaviours that may result in more exposure to contaminated water. We also assume that age modifies the relationship between past HEV infection and acute jaundice as some evidence suggests children may be more likely to be asymptomatic than adults [S2]. We assume that travel does not relate to drinking water source in our study due to the difference in time scales with only 7% of people in our study reporting having left the village in the past year and drinking water sources relating to usage in week prior to the survey.

***
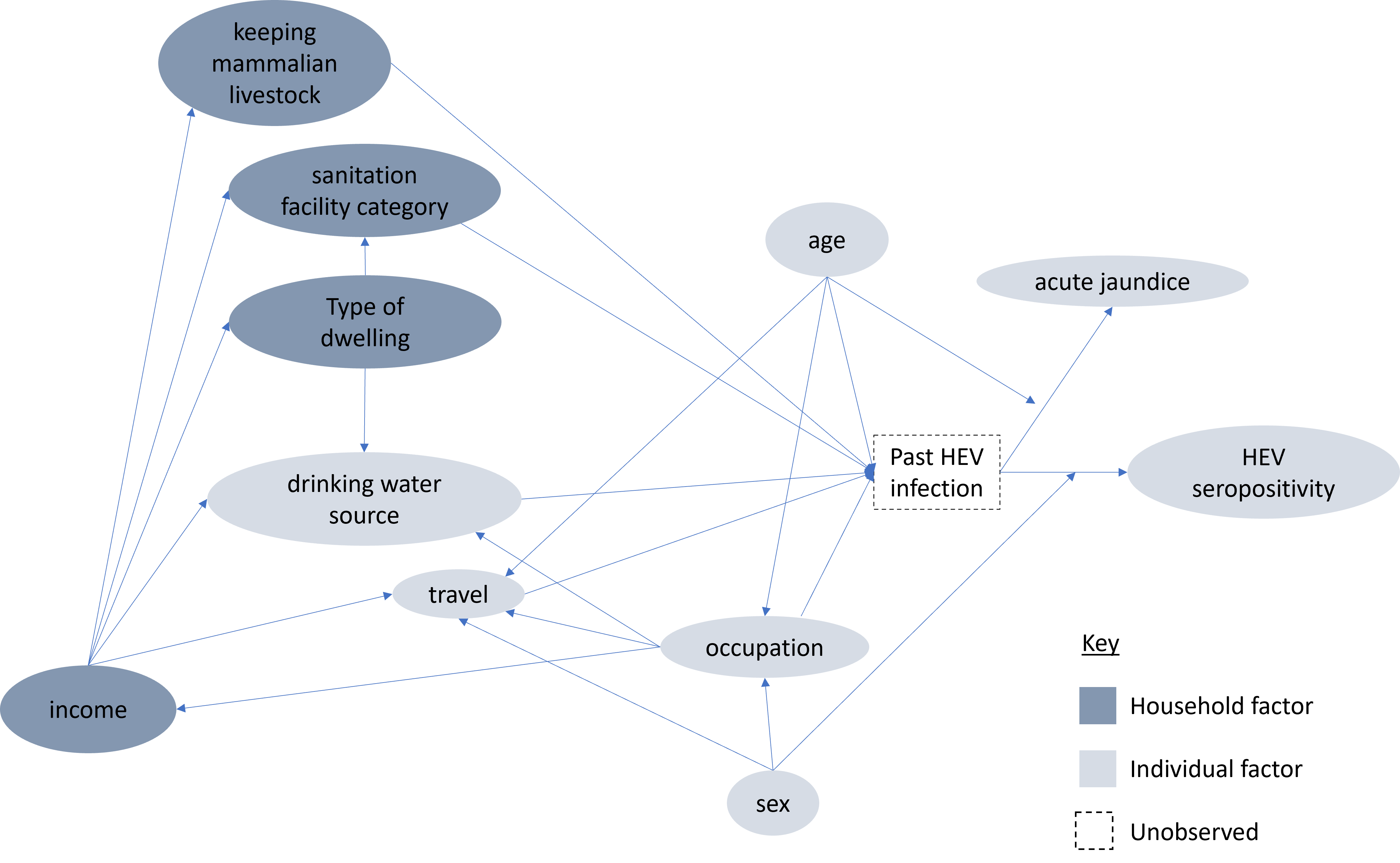
***

***Figure S2.*** *Directed Acyclic Graph* *representing the causal relationships between potential risk factors and HEV seropositivity, as assumed* a priori*. An arrow from a factor to an arrow signifies that factor as a modifier of the relationship represented by the arrow.*

***Table S1.*** *Number of individuals and households sampled during the study.*

|  | R1: Mar-Jun 2021 | | R2: Jan-Feb 2022 | | Both rounds | |
| --- | --- | --- | --- | --- | --- | --- |
|  | individuals | households | individuals | households | individuals | households |
| Participated | 2337 | 580 | 2038 | 494 | 1993 | 494 |
| Blood sampled | 2308 | 580 | 1986 | 493 | 1862 | 493 |
| HEV result available | 2301 | 580 | 1986 | 493 | 1856 | 492 |
| Excluding borderline results | 2294 | 579 | 1978 | 492 | 1846 | 491 |

*Note that one household enrolled at round 1 had split into two new households by r2, but this table still counts them as a single household followed up.*


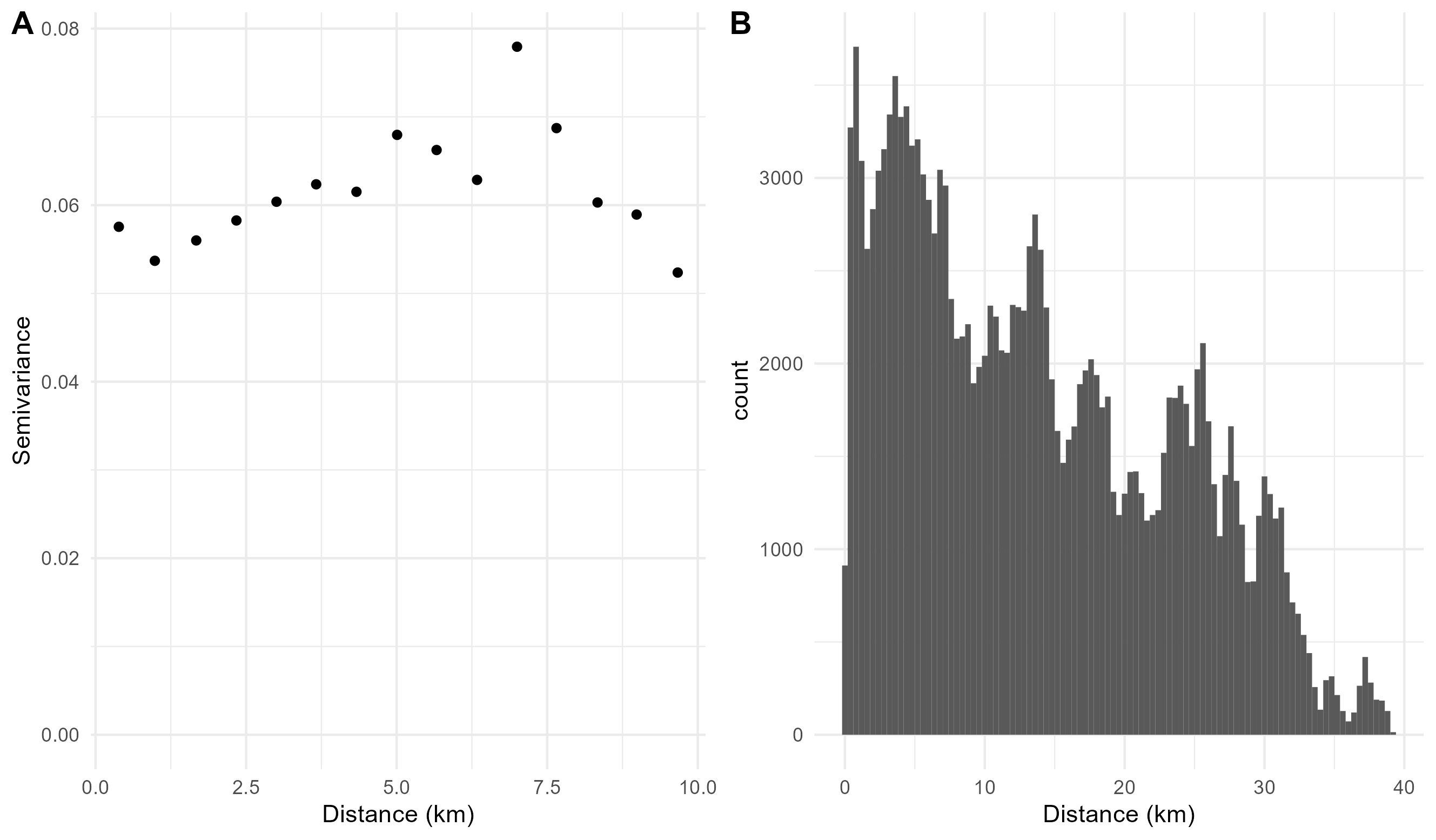


***Figure S3: A****. The semivariogram showing the semivariance in household level seroprevalence plotted against the distance between households.* ***B.*** *The distribution of distance between sampled households.*

***Table S2****: demographic characteristics of study participants, shown overall, split by sex and for the community within the cluster of high seroprevalence in southeast Sitakunda.*

|  | **Characteristic** | | **all tested**  **n (%)** | **male**  **n (%)** | **female**  **n (%)** | **cluster***  **n (%)** |
| --- | --- | --- | --- | --- | --- | --- |
| Individual level | Sex | Female | 1232 (54%) | NA | 1232 (100%) | 65 (47%) |
|  |  | Male | 1062 (46%) | 1062 (100%) | NA | 72 (53%) |
|  | Age in years | <5 | 93 (4%) | 53 (5%) | 40 (3%) | 6 (4%) |
|  |  | 5-14 | 436 (19%) | 213 (20%) | 223 (18%) | 23 (17%) |
|  |  | 15-39 | 1029 (45%) | 444 (42%) | 585 (47%) | 73 (53%) |
|  |  | 40-100 | 736 (32%) | 352 (33%) | 384 (31%) | 35 (26%) |
|  | Occupation | Homeworker | 844 (37%) | 78 (7%) | 766 (62%) | 47 (34%) |
|  |  | Business* | 502 (22%) | 438 (41%) | 64 (5%) | 42 (31%) |
|  |  | Farmer | 80 (3%) | 79 (7%) | 1 (0%) | 6 (4%) |
|  |  | Student | 666 (29%) | 345 (32%) | 321 (26%) | 30 (22%) |
|  |  | Child | 96 (4%) | 51 (5%) | 45 (4%) | 4 (3%) |
|  |  | None | 71 (3%) | 41 (4%) | 30 (2%) | 2 (1%) |
|  |  | Other | 34 (1%) | 29 (3%) | 5 (0%) | 6 (4%) |
|  |  | NA | 1 (0%) | 1 (0%) | NA | NA |
|  | Travel: time since last leaving the village | > 1 year | 2140 (93%) | 982 (92%) | 1158 (94%) | 129 (94%) |
|  |  | 1 month - 1 year | 112 (5%) | 59 (6%) | 53 (4%) | 5 (4%) |
|  |  | 1 week - 1 month | 24 (1%) | 10 (1%) | 14 (1%) | 3 (2%) |
|  |  | < 1 week | 18 (1%) | 11 (1%) | 7 (1%) | NA |
|  | Primary drinking water source was unavailable at least once in the past month | No | 2199 (96%) | 1019 (96%) | 1180 (96%) | 108 (79%) |
|  |  | Yes | 93 (4%) | 41 (4%) | 52 (4%) | 28 (20%) |
|  |  | NA | 2 (0%) | 2 (0%) | NA | 1 (1%) |
|  | Water source category | improved | 2287 (100%) | 1058 (100%) | 1229 (100%) | 136 (99%) |
|  |  | unimproved | 3 (0%) | 1 (0%) | 2 (0%) | NA |
|  |  | NA | 4 (0%) | 3 (0%) | 1 (0%) | 1 (1%) |
|  | Use of piped water in the past week | No | 1981 (86%) | 910 (86%) | 1071 (87%) | 105 (77%) |
|  |  | Yes | 309 (14%) | 149 (14%) | 160 (13%) | 31 (23%) |
|  |  | NA | 4 (0%) | 3 (0%) | 1 (0%) | 1 (1%) |
|  | Use of tubewell in the past week | No | 466 (20%) | 225 (21%) | 241 (20%) | 40 (29%) |
|  |  | Yes | 1824 (80%) | 834 (79%) | 990 (80%) | 96 (70%) |
|  |  | NA | 4 (0%) | 3 (0%) | 1 (0%) | 1 (1%) |
|  | Use of water from a public tap/standpipe in the past week | No | 2109 (92%) | 971 (91%) | 1138 (92%) | 128 (93%) |
|  |  | Yes | 181 (8%) | 88 (8%) | 93 (8%) | 8 (6%) |
|  |  | NA | 4 (0%) | 3 (0%) | 1 (0%) | 1 (1%) |
|  | Self-reported ever having had acute jaundice | No | 1829 (80%) | 842 (79%) | 987 (80%) | 105 (77%) |
|  |  | Yes | 56 (2%) | 33 (3%) | 23 (2%) | 1 (1%) |
|  |  | NA | 409 (18%) | 187 (18%) | 222 (18%) | 31 (23%) |
| Household level | Type of dwelling | Single house | 1509 (66%) | 712 (67%) | 797 (65%) | 123 (90%) |
|  |  | Several separate structures | 311 (14%) | 137 (13%) | 174 (14%) | 14 (10%) |
|  |  | Flat in a multi-story building | 210 (9%) | 94 (9%) | 116 (9%) | NA |
|  |  | Flat in a single-story building | 166 (7%) | 74 (7%) | 92 (7%) | NA |
|  |  | Room in a larger dwelling | 98 (4%) | 45 (4%) | 53 (4%) | NA |
|  | Household income | <10,000 BDT | 367 (16%) | 166 (16%) | 201 (16%) | 31 (24%) |
|  |  | >10,000 BDT | 1927 (84%) | 896 (84%) | 1031 (84%) | 105 (76%) |
|  | Sanitation facility category | Improved private | 1736 (76%) | 800 (75%) | 936 (76%) | 87 (64%) |
|  |  | Improved shared | 543 (24%) | 255 (24%) | 288 (23%) | 50 (36%) |
|  |  | Unimproved | 15 (1%) | 7 (1%) | 8 (1%) | NA |
|  | Keeps mammalian livestock in the household | No | 1631 (71%) | 756 (71%) | 875 (71%) | 74 (54%) |
|  |  | Yes | 658 (29%) | 305 (29%) | 353 (29%) | 63 (46%) |
|  |  | NA | 5 (0%) | 1 (0%) | 4 (0%) | NA |

**the subset of households located in the high seroprevalence cluster in south-east Sitakunda*


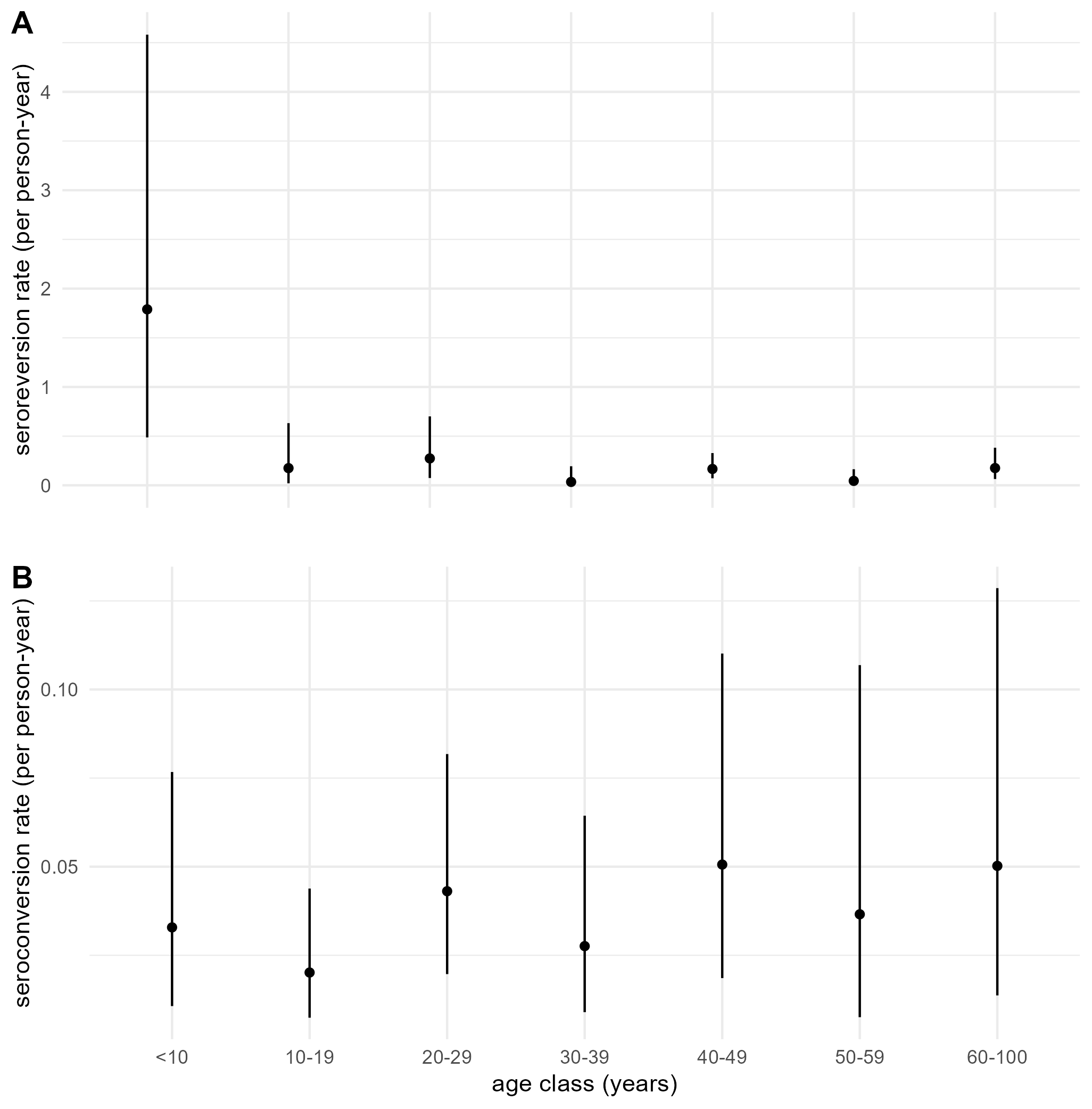


***Figure S4.*** *Annual seroconversion and seroreversion rates by age.* ***A.*** *the annual seroconversion rate per capita by age shown with 95%CIs, accounting for household sampling.* ***B.*** *The annual seroreversion rate per capita by age shown with 95%CIs, accounting for household sampling.*


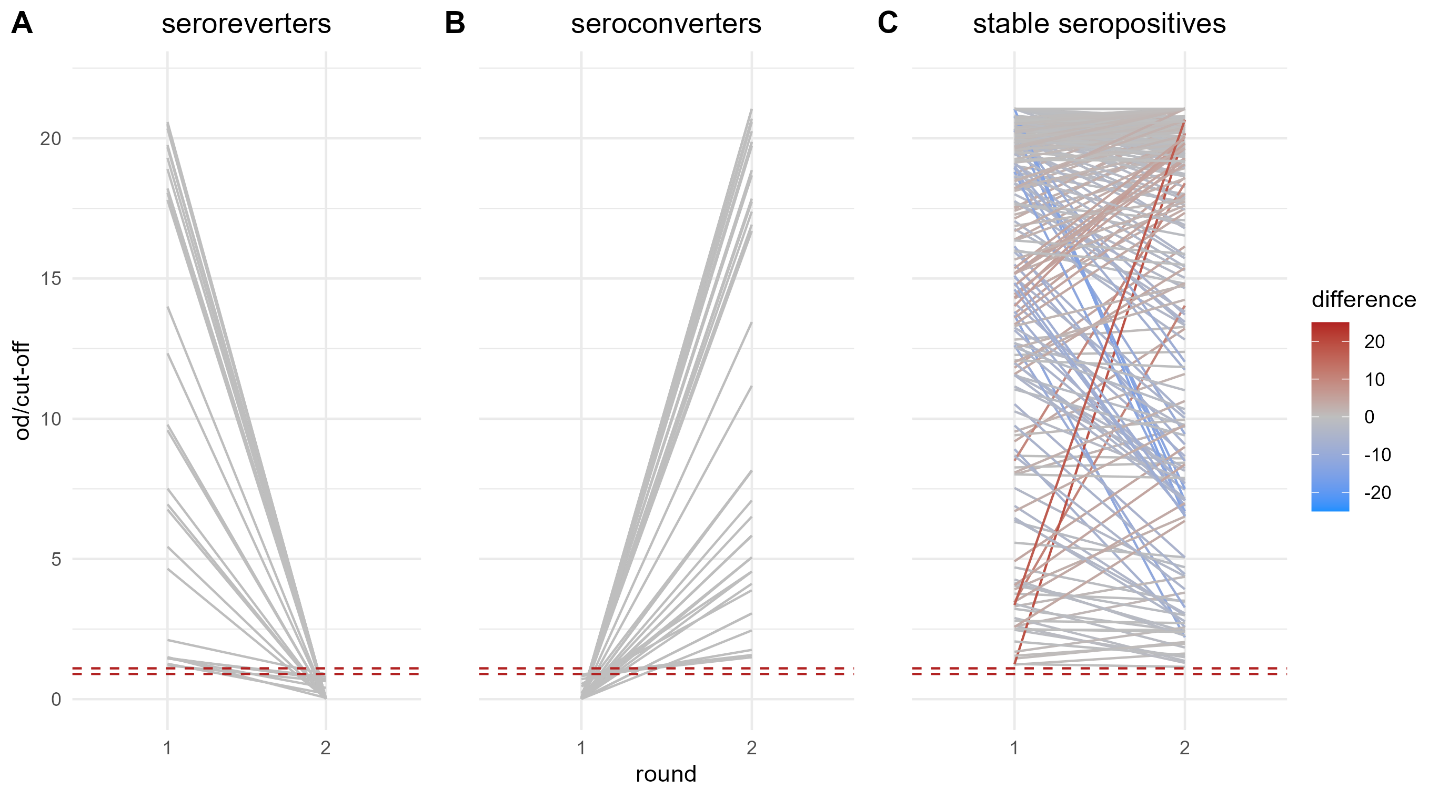


***Figure S5****. The changes in the optical density (od) to cut-off ratios for samples taken at baseline and at follow-up from* ***A.*** *individuals who seroreverted during the study,* ***B.*** *individuals who seroconverted during the study, and* ***C****. Individuals who were classed as seropositive at both time points. The area below dashed red lines shows values classed as seronegative, and the area above dashed red lines shows values classed as seropositive, whilst the values between the dashed red lines are borderline and have been excluded.*

***Table S3.*** *The difference in expected log predicted density (ELPD) compared to the preferred catalytic model of seroconversion, as estimated using Leave One Out Cross-validation*

| ELPD difference (SE) | |  |
| --- | --- | --- |
| 1. Constant FoI | 1. Age-varying FoI |  |
| -22.3 (9.5) | -3.2 (6.6) | Model 1  No seroreversion (*ρ* = 0) |
| -72.4 (0.5) | 0.0 (0.0) | Model 2  *ρ* fixed based on longitudinal data |
| -23.4 (9.6) | -1.7 (5.2) | Model 3  *ρ* fitted to cross-sectional data |


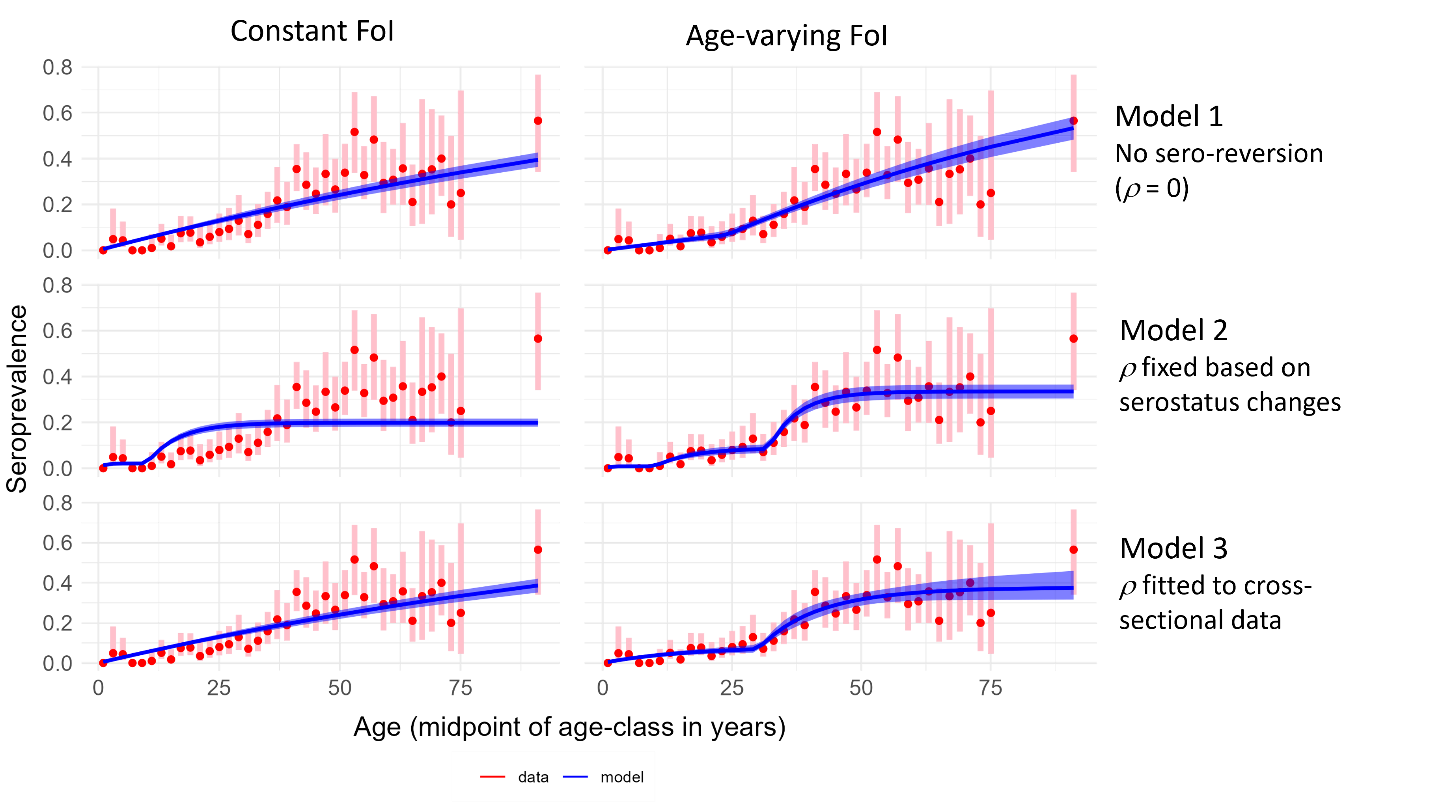


***Figure S6.*** *Predicted seroprevalence by age. Predicted seroprevalence by age is shown in blue for each model, overlaid on the age-stratified baseline seroprevalence measured in our dataset and plotted at the mid-point of each age class. Blue transparent ribbon shows 95%Credible intervals and pink error bars show 95%Confidence intervals for the data.*

***
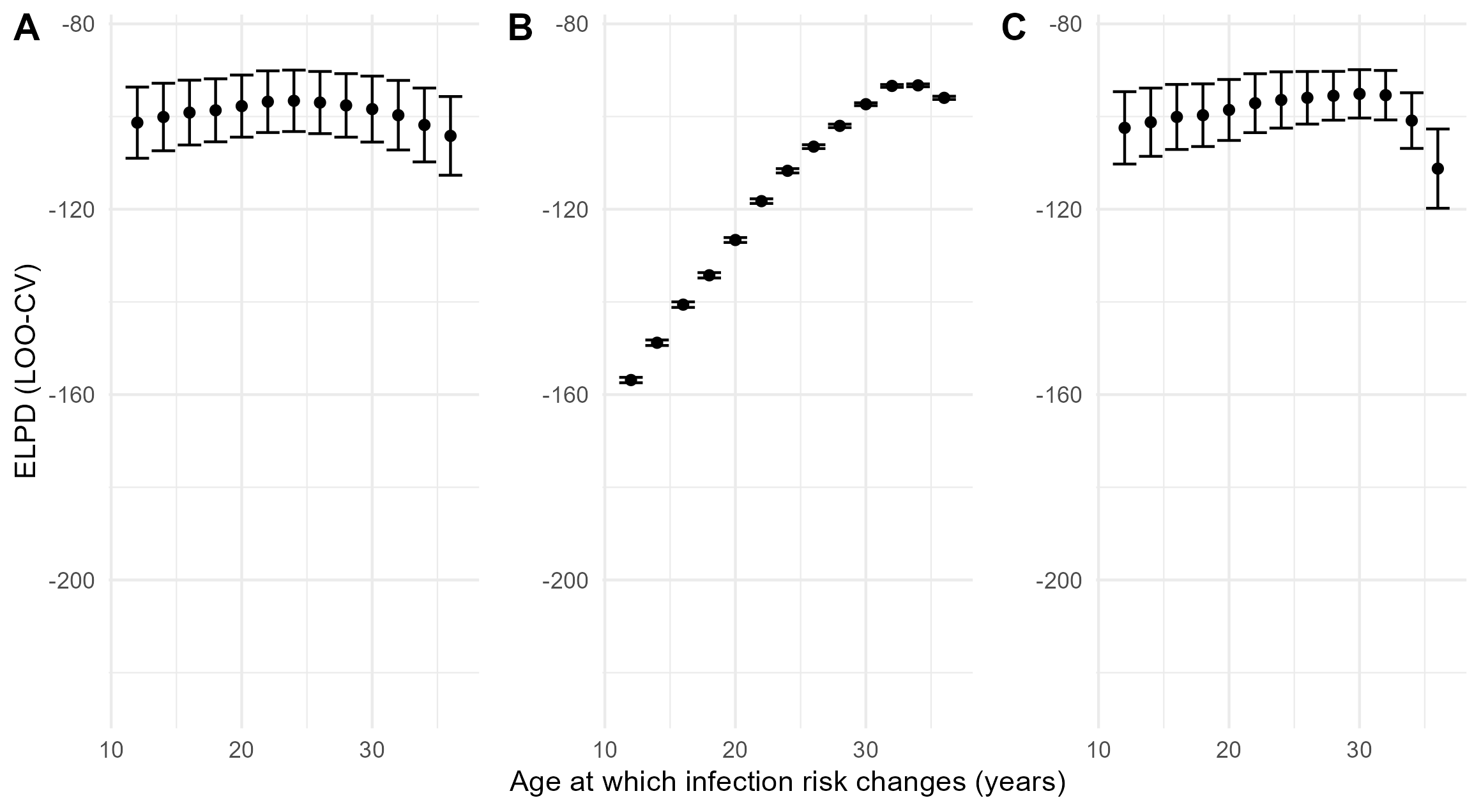
Figure S7.*** *The expected log-predictive density (ELPD) for different age thresholds for changing risk of infection. The EPLD as estimated using LOO-CV is shown with standard error bars for* ***A.*** *Model 1 in which we assume antibodies last for life (no seroreversion),* ***B.*** *Model 2 assuming instead that the rate of seroreversion that we measured empirically in our cohort,* *and* ***C****. Model 3 in which the annual risk of infection and the rate of seroreversion are both simultaneously fitted to cross-sectional seroprevalence data.*

***
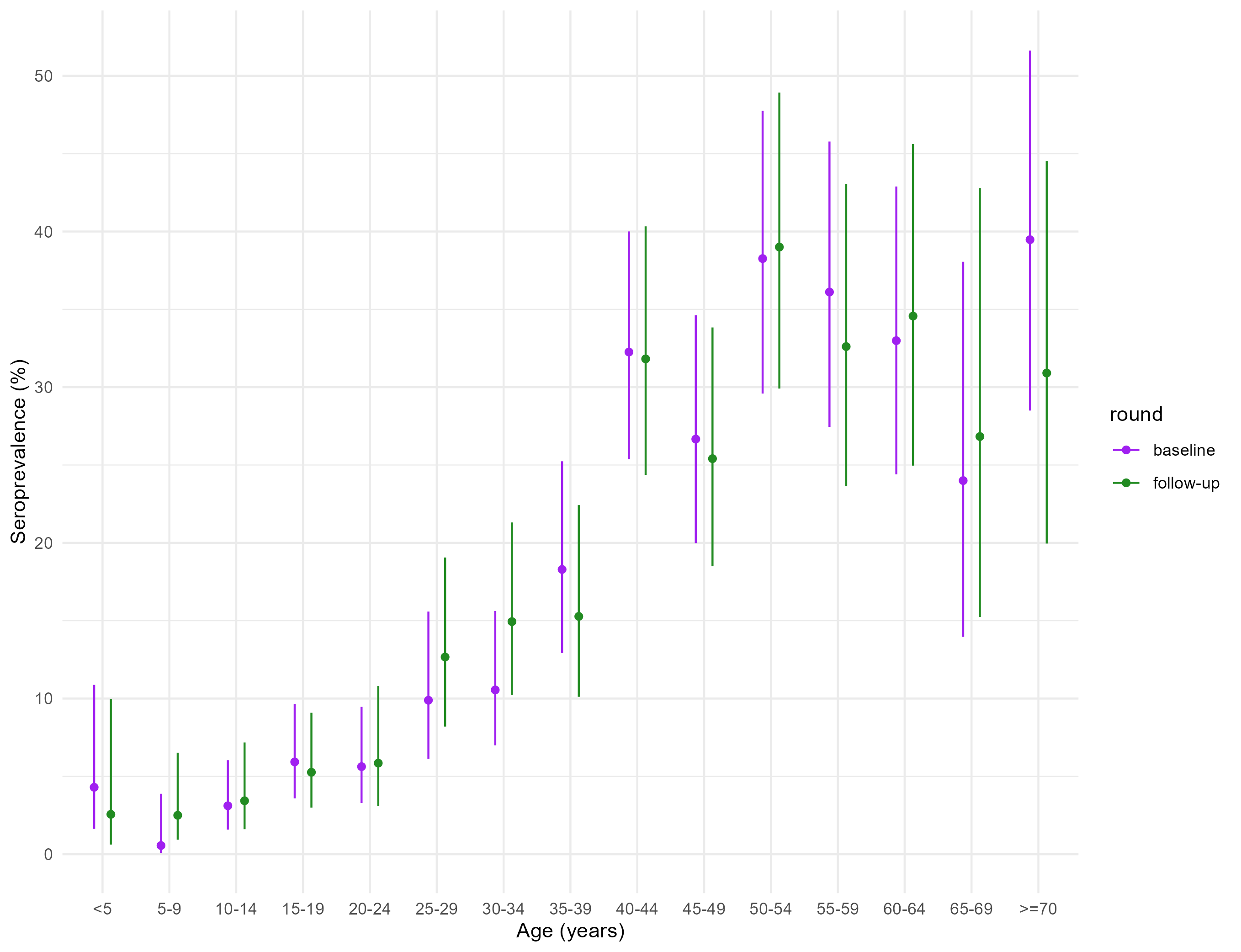
***

***Figure S8.*** *A comparison of the age-stratified seroprevalence by round.*

***Table S4.*** *A comparison of annual risk of infection estimates when using age-stratified seroprevalence at baseline versus at follow-up.*

| Round | Annual risk of infection (mean and 95% CrI) | |
| --- | --- | --- |
|  | <30 year olds | >30 year olds |
| Baseline | 1.1% (0.8-1.4%) | 4.5% (3.7-5.4%) |
| Follow-up | 0.9% (0.7, 1.2%) | 4.1% (3.3-5.1%) |

***
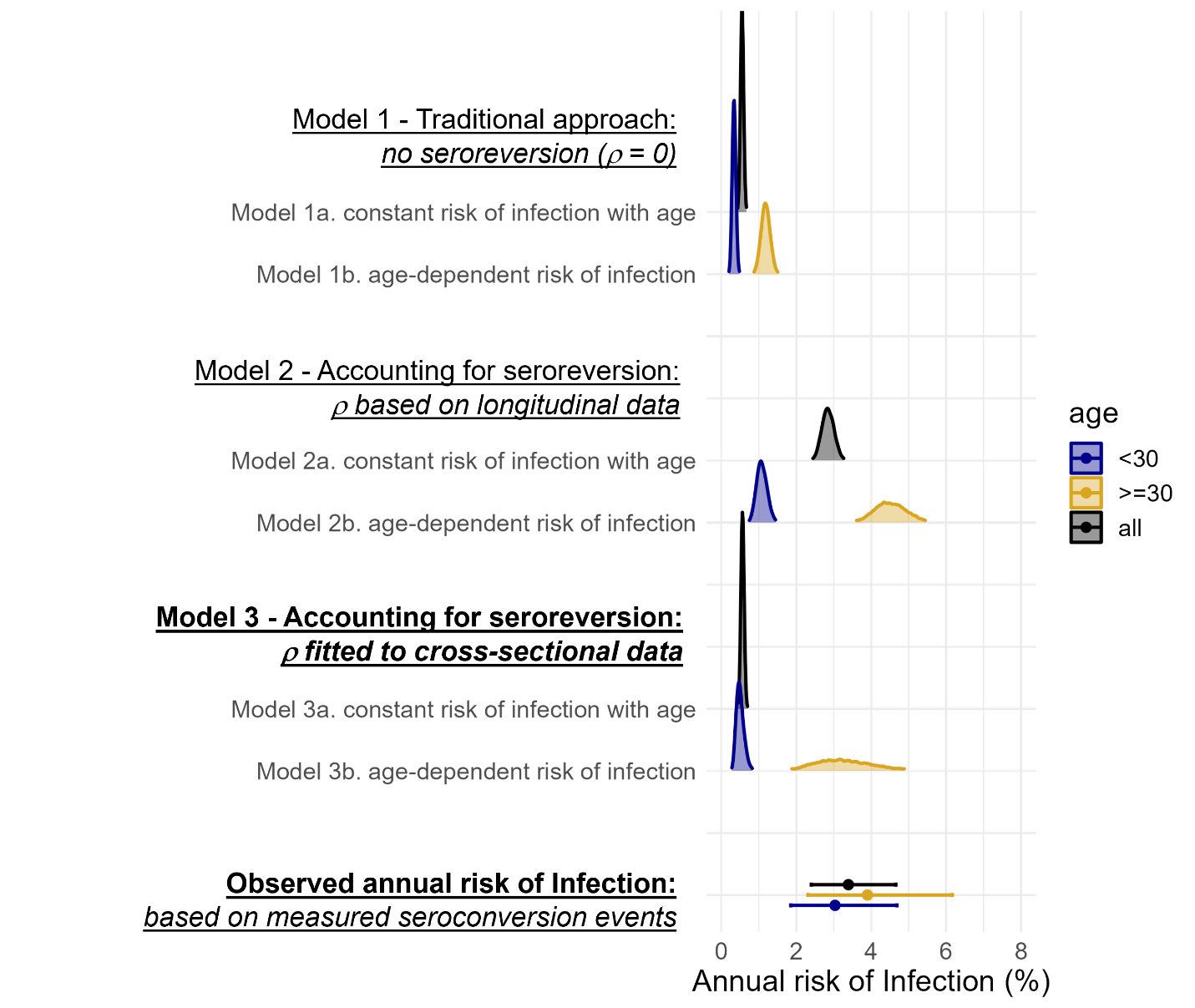
***

***Figure S9.*** *A comparison of posterior estimates of the annual risk of infection when we fit the rate of seroreversion, ρ, simultaneously with the annual risk of infection, to age-stratified cross-sectional seroprevalence data. The results are shown alongside Models 1 and 2 (presented in the main text), along with the estimates of the annual risk of infection from observed seroconversion events captured in our longitudinal serostatus data (points with bars representing 95%CIs.*

***Additional references cited in the Supplementary Materials***

S1. **Fink AL, Klein SL**. (2018) The evolution of greater humoral immunity in females than males: implications for vaccine efficacy. *Current opinion in physiology*; **6**: 16–20.

S2. **Verghese VP, Robinson JL**. (2014) A Systematic Review of Hepatitis E Virus Infection in Children. *Clinical Infectious Diseases*; **59**: 689–697.
